# Decreased Size of Mammary Tumors Caused by Preoperative Treatment with Aglepristone in Female Domestic Dogs (*Canis familiaris*) Do Not Influence the Density of the Benign Neoplastic Tissue Measured Using Shear Wave Elastography Technique

**DOI:** 10.3390/ani11020527

**Published:** 2021-02-18

**Authors:** Barbara Pieczewska, Kamila Glińska-Suchocka, Wojciech Niżański, Michał Dzięcioł

**Affiliations:** 1 Department of Reproduction and Clinic of Farm Animals, Wroclaw University of Environmental and Life Sciences, 50-366 Wrocław, Poland; barbara.pieczewska@upwr.edu.pl (B.P.); wojciech.nizanski@upwr.edu.pl (W.N.); 2 Department of Internal Medicine and Clinic of Diseases of Horses, Dogs and Cats, Wrocław University of Environmental and Life Sciences, 50-366 Wrocław, Poland; kamila.glinska-suchocka@upwr.edu.pl

**Keywords:** dogs, mammary tumors, aglepristone, shear wave elastography, SWE

## Abstract

**Simple Summary:**

Shear wave elastography (SWE) is a new diagnostic method allowing for a non-invasive differential diagnosis between benign and malignant mammary tumors. Mammary tumors in dogs are often hormone-dependent, and a surgical approach is currently the most recommended method for the treatment of mammary neoplasia. However, preoperative, pharmacological therapy eliminating hormonal stimulation can sometimes be indicated to reduce the size of the tumor and facilitate subsequent surgery. The aim of this study was to evaluate the influence of the reduction in the benign tumor size observed after treatment with the use of aglepristone on the shear wave elastography results, obtained before and after therapy. We noticed that a fast and significant reduction in tumor size, as a consequence of preoperative pharmacological treatment, did not influence the diagnosis performed using the SWE technique. These results seem to indicate the accuracy and usefulness of this new diagnostic method, and confirm the hypothesis that tumor tissue density is a valuable parameter when determining the benignancy of mammary tumors in dogs.

**Abstract:**

Shear wave elastography (SWE) can be useful to discriminate between malignant and benign mammary tumors. In dogs with elevated progesterone levels compared to the baseline and fast-growing tumors, treatment with the use of aglepristone allows for tumor size reduction, which facilitates surgery. This study aimed to evaluate the influence of the preoperative treatment of benign mammary tumors (BMTs), performed with the use of aglepristone, on the density of the tumor tissue measured by SWE. Twelve female dogs with diagnosed BMTs and increased levels of progesterone were treated with aglepristone (Alizine, Virbac, France) at 10 mg/kg s.c. (Subcutaneous injection). twice, with a 24 h interval. The density of the tumor was evaluated by SWE before and after the treatment. The type of tumor was evaluated by fine needle aspiration cytology before treatment, and a histopathological examination was made after surgical removal, performed after the aglepristone treatment. In all the cases, a significant reduction in the mammary tumor’s size was observed following treatment, with no influence on the density of the tumor’s tissue measured by SWE. Similar studies on malignant mammary tumors are warranted to verify if in these cases, density will also be a constant parameter that is not dependent on the tumor size.

## 1. Introduction

Canine mammary tumors (CMTs) are the most frequently diagnosed neoplasms in female dogs, constituting approximately 42% of all neoplasms and 82% of reproductive system neoplasms [1,2,3,4].

Although mammary tumors are primarily treated surgically [1,2,5,6,7], some methods of pharmacological treatment or treatment preceding surgical intervention have been described in the literature [2]. Fast-growing tumors in dogs are usually observed in the metoestrus period, whereby long periods of elevated levels of progesterone strongly stimulate the growth of the tumor tissue, which often has progesterone receptors [8]. Observations of this mechanism allow for the application of competitive inhibitors of progesterone receptors for the pharmacological treatment of some mammary tumors in dogs [5,9,10].

The use of an ultrasound examination of the tumor can bring to light useful information regarding the morphology of the lesion. If the Doppler technique is also applied, the quantitative analysis of tumor perfusion can be performed [11,12,13]. Although the Doppler technique has been used to differentiate between malignant and benign breast lesions [14], ultrasound elastography (USE), which allows for quantitative assessment of tissue stiffness, seems to be currently the most valuable imaging technique for this purpose [15]. Among different elastographic techniques, shear wave elastography (SWE) is described as the most promising tool allowing for fast, non-invasive prediction of the malignancy of the tumors, based on the evaluation of the tissue weaving density [15,16,17,18,19,20]. It is a medical imaging method that maps the elastic properties and stiffness of soft tissue within a given organ, characteristics that often change in compromised tissue.

Although the usefulness of SWE in mammary tumor diagnosis has been confirmed in dogs, there are no available data concerning the existence of factors that could change the stiffness and density of the examined tissues, which has already been described in human medicine [17,18,21,22]. Thus, the aim of this study was to evaluate the influence of the use of the aglepristone (a competitive inhibitor of progesterone receptors) as a preoperative treatment on tumor tissue density as a parameter of mammary tumor differentiation, evaluated by shear wave elastography in the female domestic dog (*Canis familiaris*) benign tumor model.

## 2. Material and Methods

### 2.1. Ethics Statement

The research was carried out in accordance with regulations on animal experimentation, and the guidelines for the use of animals in research. The Local Institutional Animal Care and Use Committee issued a statement approving the experimental protocol (resolution no. 26/2020).

### 2.2. Location of Animals and Their Selection

The study was conducted in the Department of Internal Diseases and in the Department of Reproduction, Faculty of Veterinary Medicine, Wrocław University of Environmental and Life Sciences.

Twelve female dogs of different breeds with an average age of 6 to 10 years which were patients of the Clinic of Reproduction were used for the study. All the females had clinically diagnosed tumors localized in the area of the mammary gland. No lung metastases in thoracic radiographs were found in any of the females, and the general condition as well as the results of biochemical tests indicated the possibility of postponing surgery and introducing preoperative therapy with aglepristone. Only non-spayed bitches with elevated levels of progesterone (above 5 ng/mL) were included in the experimental group.

### 2.3. Progesterone Level Evaluation

The progesterone concentration in the peripheral blood in the females was determined by an enzyme-linked fluorescence assay (ELFA; mini VIDAS® Biomerieux, Marcy-l’Étoile, France) with coefficients of variation (CV) for the mean level of progesterone 21, 66 ng/mL at a level of 3.8% [23].

### 2.4. Aglepristone Administration

Animals with diagnosed tumors and with an elevated level of progesterone were treated with double subcutaneous injections of aglepristone (RU 46534) (Alizine, Virbac, France) administered at 10 mg/kg, at an interval of 24 h.

### 2.5. Histopathological Evaluation and Shear Wave Elastography of the Tumors 

The tumors were initially evaluated on the basis of fine needle aspiration cytology (FNAC). After surgical removal of the tumors, a histopathological examination was performed [17,18,24,25]. There were two examinations performed with the use of SWE. The first one was done simultaneously with the FNAC before treatment, and the second one took place after the pharmacological treatment, before surgical tumor removal.

The fine needle aspiration biopsy of the mammary gland tumor was performed using a 0.9 mm diameter 20 G needle attached to a 10 mL syringe. The collected material was spread onto a glass slide and a cytological smear was made. The smears were fixed by Cytofix® (Samko, Dobczyn, Poland) and sent to the Cytopathology Lab, where it was stained with hematoxylin and eosin (H&E) as standard staining and its evaluation was performed according to the classification from the WHO for canine mammary gland tumors.

The histopathological examination was performed by an experienced pathologist, and histopathological classification follows the classification from the WHO for canine mammary gland tumors. Sections of tumors were fixed in 7% neutral-buffered formalin solution. After trimming, the samples were embedded in paraffin, and from each paraffin block, a slide with 2 µm thickness was cut. Slides were stained with H&E as standard staining.

Shear wave elastography (SWE) was performed with the use of the Aixplorer® ultrasound system (SuperSonic Imagine, Aix en Provence, France). It was carried out on unsedated animals in dorsal recumbency according to the protocol described by Glińska-Suchocka [18], where tumors were classified as benign when a value of tissue elasticity below 80 kPa was noted. In each patient, five measurements of the tissue elasticity were taken, and an average value was calculated. 

### 2.6. Tumor Size Evaluation

The measurements of the mammary tumors were performed before and after treatment. In the experiment, only females affected by a single tumor were classified. Tumor measurements were performed with the use of a traditional caliper, which allows for obtaining two-dimensional measurements, taking into account the highest values of the width and length of the tumor. Based on obtained data, an approximate area value of the tumors was calculated.

### 2.7. Statistical Methods

The paired student’s *t*-test was used to compare the mean values of the mammary tumor hardness (kPa) and the tumor area (mm^2^) between the groups before and after treatment. The analysis was performed at the 5% significance level using the STATISTICA package (data analysis software system), version 13 by TIBCO Software Inc. (2017) (Palo Alto, CA, USA).

## 3. Results

**Figure 1:**
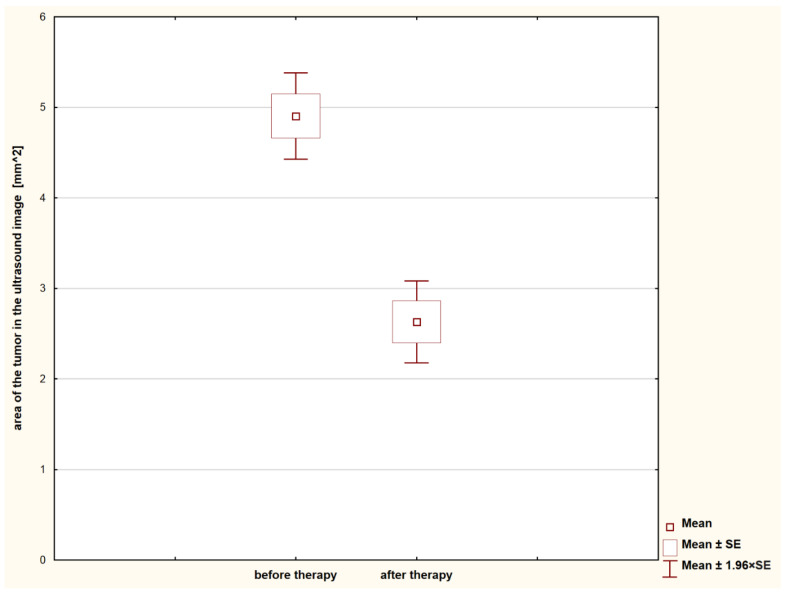
Reduction of the tumor size observed in individual females after treatment with aglepristone. SE = Standard error.

**Figure 2:**
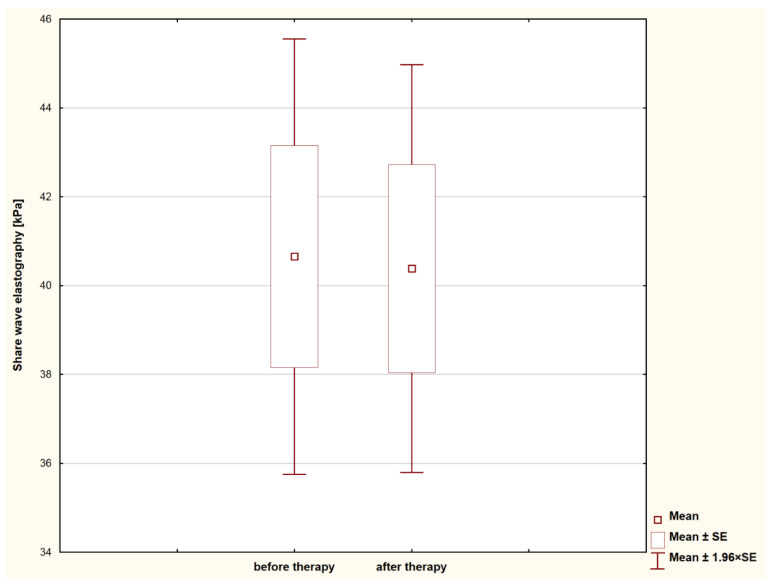
Statistical evaluation of the tissue density of the benign tumors treated with the use of aglepristone, before and after treatment. SE = Standard error.

Several different types of benign tumors were classified in the study (Table 1). In all the cases, the administration of the double doses of aglepristone gave a significant, visual reduction in the tumor size observed after approximately 14 days (*p* < 0.001; Figure 1). The percentage of tumor size reduction reached from 33 to 65% (46.5% in average). Despite that, there was no statistically significant difference between tissue density in the examined lesions before and after aglepristone treatment (*p* = 0.525; Figure 2). Thus, tumors diagnosed as benign using SWE examination and fine needle aspiration cytology before aglepristone treatment were also visible during the second SWE examination and were confirmed by histopathological examination of surgically removed lesions (Table 1). In all the studied cases, the first and the second examination yielded the same results regarding the benignancy of the examined tumors, despite the rapid change in the tumor size.

## 4. Discussion

Canine mammary tumors are regarded as an animal model for human breast cancer [2,26,27,28]. However, important differences can be found, particularly in the treatment of these tumors between the two species [7,29]. Chemotherapy along with surgery are standard procedures in breast cancer management in humans, while chemotherapy is not routinely administered in dogs [2,7]. Much more popular in dogs is a surgical approach achieved by performing lumpectomy (removal of the tumor alone), simple mastectomy (removal of only the affected gland), modified radical mastectomy (removal of the affected gland, together with glands sharing lymphatic drainage and associated lymph nodes) and radical mastectomy (removal of the entire mammary chain with associated lymph nodes) [6,30]. However, in some cases of invasive malignant mammary gland tumors, surgery alone does not produce satisfactory results [31].

Antimetabolic drugs, alkylating agents, antitumor antibiotics, phospholipids, homeopathic medications, the recombinant measles virus and many other substances are proposed for canine tumor treatment [1,2]. Nonsteroidal anti-inflammatory drugs (NSAIDs) such as cyclooxygenase (COX) inhibitors (e.g., piroxicam) and estrogen antagonists (tamoxifen) have formerly been some of the most popular agents used in the preoperative treatment of canine mammary tumors. In cases of inflammatory mammary carcinoma, NSAIDs were found to be an effective treatment [32]. In such cases, anti-inflammatory as well anti-cancer effects of piroxicam were taken into account when choosing the treatment [33,34]. It was observed that the treatment resulted in a reduction of inflammation, as well as a significant reduction in the tumor size. 

On the other hand, Mainenti et al. [8] stated that the prognostic value of hormone receptor expression in canine mammary tumors (CMTs) is much less clearly understood than in humans. The involvement of two main sex hormones, estrogens and progesterone, in the development of canine mammary tumors is unquestionable. Thus, the use of agents interacting with these hormone receptors can be used in the pharmacological treatment of mammary tumors in this species. In our study, all females included in the experiment had levels of progesterone higher than 5 ng/mL (from 16.37 ng/mL to 40.35 ng/mL, sample mean 23.87 ng/mL, st.dev. 8.87). Those results were in accordance with the data from anamnesis, suggesting that females were in the luteal phase of the ovarian cycle.

Hormone receptor blockers are highly effective in treatment, and result in a significant tumor size reduction [35,36,37,38]. In dogs, aglepristone (RU534), a progesterone receptor antagonist, has been successfully used to terminate pregnancy in bitches and treat pyometra, vaginal tumors and mammary hyperplasia [5,9,10,39]. The results obtained in our experience confirmed the literature reports. It also shows that continuous secretion of the hormones is one of the important elements influencing the progressive growth of the tumor masses. As the current evidence indicates, treatment with aglepristone is useful for the management of canine mammary tumors, and the use of this drug could become increasingly popular as a method of pretreatment, or a method of palliative treatment in cases where a surgical approach is contraindicated [5,9].

Although the size of the tumor can influence the prognosis in the context of the safety of the surgical procedure, the obtained results indicate that this parameter should not be the element influencing prognosis, since the type of tumor obviously could not be changed by the tumor size reduction, and the features of the tumors (in our experiment being stiffness of the tissue) also did not change.

Shear wave elastography (SWE) is a quantitative and non-invasive method that has been used to monitor many organs such as the liver, thyroid gland, prostate, kidney, lymph node and mammary gland [15,19,22,40,41,42,43,44,45,46,47]. It allows the measurement of tissue density, and is often used for the identification and evaluation of the quality of detected tumors. Elastography has been confirmed as an effective tool for the differentiation between benign and malignant tumors both in humans and dogs [17,18,47,48]. Quantitative stiffness data estimated in kilopascals (kPa) were significantly higher in the malignant areas compared with the benign ones, which was also observed in our experiment.

However, this diagnostic technique may provide false results, as has been reported by some authors in recent studies, suggesting that a histological diagnosis remains the gold standard for tumor identification [21,42]. According to Kim et al. [21], false SWE features were more frequently observed in benign masses and in younger patients. This observation, although obtained in a relatively small number of cases, was not confirmed in our study, since just benign tumors had been examined, and in our experiment, accuracy of the SWE diagnosis had been confirmed. Due to the fact that canine mammary tumors are characteristic of older females (which was also the case in our experiment), we cannot refer to the issue of false results in the younger bitches. 

Junker et al. [48] described the principles for the real-time elastography of the prostate, and noted that benign lesions could be difficult to distinguish from prostatitis, fibrosis, atrophy, adenomyomatosis and benign prostatic hyperplasia (BPH), as these conditions were also associated with increased tissue stiffness. Thus, the recognition of the factors potentially influencing the accuracy of the obtained SWE results seems to be important. In this context, a significant and fast reduction of the size of the tumor could potentially be considered as a factor that could influence the structure and density of the tumors. Moreover, since in our study we focused on the neoplastic changes of the mammary gland in bitches, it would be advisable to compare the chosen features (e.g., stiffness) of the tumors with other non-neoplastic changes (like acute chronic inflammation, galactostasis, etc.), and with physiological conditions like milk accumulation during lactation.

The stiffness and density of the tissue seem to be characteristic for particular tissues, and any changes in this parameter have a prognostic and diagnostic value in the detection of pathological conditions in many organs. However, in some circumstances, the physiological conditions can influence these parameters, and different values may be obtained during continuous examinations. Kennedy et al. [49] mentioned that density differences in subcutaneous abdominal adipose tissue layers were noted during pregnancy in women using elastography. In the case of the prostate, SWE examination results may depend on physiological changes, demonstrating an increasing stiffness with growing age and volume [48]. On the other hand, Chen et al. [50] stated that the stiffness of the mammary gland did not correlate with age, and there was no positive correlation between breast stiffness and breast volume. Those authors also noticed that malignant lesions exhibited high stiffness not only in the lesion but also in the surrounding tissue, whereas benign lesions demonstrated low stiffness in both the lesion and the surrounding area, which seems to be associated with a significantly higher content of collagen fiber in the malignant lesions [50]. In the context of our study, however, performed on females suffering from benign tumors, further analyses of changes in proportions of tissue components are also worth performing, as a comparison of the composition of untreated tumors and tumors treated with aglepristone, to find out which element of the neoplastic tissue is directly responsible for the tumor size reduction.

Changes in the density of the examined tissues can also result from the drugs used for the targeted treatment of particular organs. In the case of liver disorders, Ferraioli et al. [22] reported that long-term nucleos(t)ide analog treatment could reverse histologic cirrhosis, which could result in changes in liver stiffness and in SWE results. Chan et al. [51] reported that liver stiffness rapidly declined in patients treated with direct-acting antivirals (DAAs). In the case of the thyroid gland, Ruchala et al. [19] stated that stiffness in subacute thyroiditis decreased as a consequence of four to ten weeks of treatment. Changes in the stiffness of the treated tissue can be expected during tendon treatment, and an elastographic examination is a recommended method for the monitoring of this process, and for the evaluation of the therapy results [52].

Thus, the use of therapeutic agents can influence the results of SWE in addition to changes in the density and stiffness of the examined tissues also caused by physiological factors. To date, the analysis of SWE of mammary tumors in dogs has not been reported. The reduction in the size of tumors observed in our study as a result of aglepristone treatment did not cause changes in their density. Hence, it may be concluded that the diagnostic value of the measurement of the density of benign mammary gland tumors in bitches is not influenced by preoperative treatment with a progesterone receptor antagonist. Furthermore, the density of the tumor is closely related to its type (benign or malignant), and not directly influenced by its size. 

## 5. Conclusions

Based on the obtained results, we can state that shear wave elastography in bitches is not influenced by a pharmacologically forced fast tumor size reduction. However, these results, taking into account a relatively small number of cases, should be treated as preliminary, and a repeated study on a larger population is advisable. Moreover, although benign mammary neoplasms in bitches have a high incidence and comprise 80% of all tumors [3], a similar study on females suffering from malignant lesions is warranted.

## Figures and Tables

**Table 1:** Results of the cytological examination of the mammary gland neoplasms in dogs diagnosed based on fine needle aspiration cytology (FNAC) performed prior to pharmacological treatment and histopathological examination performed after pharmacological treatment and surgical removal of the lesions.

Animals	Mammary Tumor Diagnosis via Fine Needle Aspirate before Treatment with Aglepristone	Mammary Tumor Diagnosis via Histopathology after Treatment with Aglepristone
1	fibroadenoma	fibroadenoma
2	adenoma	adenoma
3	adenoma	adenoma
4	benign mixed tumor	benign mixed tumor
5	fibroadenoma	fibroadenoma
6	benign mixed tumor	benign mixed tumor
7	benign mixed tumor	benign mixed tumor
8	benign mixed tumor	benign mixed tumor
9	adenoma	adenoma
10	adenoma	adenoma
11	benign mixed tumor	benign mixed tumor
12	fibroadenoma	fibroadenoma

## Data Availability

The data that support the findings of this study are available from the corresponding author [MD], upon reasonable request.
